# Functional Characterization of the Disease-Associated N-Terminal Complement Factor H Mutation W198R

**DOI:** 10.3389/fimmu.2017.01800

**Published:** 2017-12-13

**Authors:** Marcell Cserhalmi, Barbara Uzonyi, Nicolas S. Merle, Dorottya Csuka, Edgar Meusburger, Karl Lhotta, Zoltán Prohászka, Mihály Józsi

**Affiliations:** ^1^MTA-ELTE “Lendület” Complement Research Group, Department of Immunology, ELTE Eötvös Loránd University, Budapest, Hungary; ^2^MTA-ELTE Immunology Research Group, Department of Immunology, ELTE Eötvös Loránd University, Budapest, Hungary; ^3^UMRS 1138, Cordeliers Research Center, Complement and Diseases Team, INSERM, Paris, France; ^4^3rd Department of Internal Medicine, Semmelweis University, Budapest, Hungary; ^5^Department of Nephrology and Dialysis, Academic Teaching Hospital Feldkirch, Feldkirch, Austria; ^6^MTA-SE Immunology and Hematology Research Group, Semmelweis University, Budapest, Hungary

**Keywords:** complement dysregulation, factor H, C3 glomerulopathy, atypical hemolytic uremic syndrome, mutation, kidney disease

## Abstract

Dysregulation of the complement alternative pathway is involved in the pathogenesis of several diseases, including the kidney diseases atypical hemolytic uremic syndrome (aHUS) and C3 glomerulopathy (C3G). In a patient, initially diagnosed with chronic glomerulonephritis, possibly C3G, and who 6 years later had an episode of aHUS, a heterozygous missense mutation leading to a tryptophan to arginine exchange (W198R) in the factor H (FH) complement control protein (CCP) 3 domain has previously been identified. The aim of this study was to clarify the functional relevance of this mutation. To this end, wild-type (FH1–4_WT_) and mutant (FH1–4_W198R_) CCPs 1–4 of FH were expressed as recombinant proteins. The FH1–4_W198R_ mutant showed decreased C3b binding compared with FH1–4_WT_. FH1–4_W198R_ had reduced cofactor and decay accelerating activity compared with the wild-type protein. Hemolysis assays demonstrated impaired capacity of FH1–4_W198R_ to protect rabbit erythrocytes from human complement-mediated lysis, and also to prevent lysis of sheep erythrocytes in human serum induced by a monoclonal antibody binding in FH CCP5 domain, compared with that of FH1–4_WT_. Thus, the FH W198R exchange results in impaired complement alternative pathway regulation. The heterozygous nature of this mutation in the index patient may explain the manifestation of two diseases, likely due to different triggers leading to complement dysregulation in plasma or on cell surfaces.

## Introduction

Complement alternative pathway dysregulation is implicated in the pathogenesis of several diseases, including the renal diseases C3 glomerulopathy (C3G) and atypical hemolytic uremic syndrome (aHUS) (1, 2). C3G is an umbrella term that covers a spectrum of clinically heterogeneous rare kidney diseases characterized by common underlying pathogenic mechanisms; its two main subclasses are dense deposit disease and C3 glomerulonephritis, characterized by deposition of C3 fragments in the glomeruli but differentiated based on the presence of electron dense deposits in the case of dense deposit disease upon electron microscopical analysis (3). aHUS is a thrombotic microangiopathy (TMA) characterized by erythrocyte fragmentation, low platelet count, and acute kidney failure (4–6).

These distinct disease entities have partly common underlying genetic and acquired susceptibility factors, and thus, shared pathological mechanism related to complement dysfunction (2, 7, 8). C3G is most commonly associated with C3 nephritic factor, an autoantibody that stabilizes the C3 convertase (C3bBb) and thus leads to enhanced complement activation (9, 10). In addition, anti-factor H (FH) autoantibodies that bind the N terminus of FH and prevent the FH-mediated fluid-phase regulation of C3bBb (11–13), and anti-factor B and anti-C3b autoantibodies that stabilize the C3bBb convertase, are described in C3G (10, 14–16). Similarly, C5 nephritic factors stabilize the C5 convertase and are associated with C3G (17). More rarely, genetic disease predisposing factors are identified in C3G patients. These include complement factor H (*CFH*) risk variants and mutations (18–22), *CFHR1* mutation (23), *CFHR5* mutation (24), and hybrid *CFHR3::CFHR1, CFHR2::CFHR5, CFHR5::CFHR2*, and *CFHR1::CFHR5* genes (25–28). In aHUS, variations of several complement genes have been described as susceptibility factors, among which FH variations being the most common (in ~30% of the patients) (5). In addition, FH autoantibodies may be present in aHUS and are strongly associated with the deletion of the *CFHR1* gene (29–31).

Factor H is the main soluble regulatory protein of the alternative pathway (32–34). FH inhibits the activation of the alternative pathway by three mechanisms: by acting as a cofactor for the serine protease factor I (FI) in the enzymatic cleavage and thus inactivation of C3b (“cofactor activity”), by preventing the assembly of the C3bBb C3 convertase enzyme, and by accelerating the decay of this convertase if already formed (“decay accelerating activity”) (35–37). FH does not only act in the fluid phase, i.e., in body fluids, but it can also bind to and protect host cells and surfaces from complement attack [reviewed in Ref. (32)]. FH is composed of 20 complement control protein (CCP) domains. The C-terminal CCP19–20 domains allow FH to recognize host surfaces under complement attack, i.e., deposited C3b in the context of host polyanionic molecules, such as sialic acid (38). The FH N-terminal CCP1–4 domains mediate binding to C3b, cofactor activity for FI, and acceleration of the decay of the alternative pathway C3bBb convertase (39).

Mutations in FH may lead to quantitative or qualitative FH deficiency and are associated with diseases (40). Some of the mutations cause secretion defect or early stop codons and thus result in truncated or undetectable FH protein in plasma (41, 42). Other mutations cause functional impairment in otherwise normally secreted FH (20, 40). Similarly, autoantibodies binding primarily in the main functional domains inhibit either the regulatory or the host cell/surface binding functions of FH [reviewed in Ref. (10)]. In general, mutations and autoantibodies affecting the N-terminal regulatory domains of FH cause alternative pathway dysregulation in plasma, leading to plasma C3 consumption and deposition of C3 fragments in the glomeruli, and are associated with C3G. On the other hand, mutations and autoantibodies interfering with the C-terminal host recognition domains of FH usually cause complement dysregulation on surfaces, such as the glomerular endothelium and basement membrane, and are associated with aHUS (2, 5, 9, 32).

Most functionally characterized FH mutations to date were those affecting the C terminus and are associated with aHUS. Relatively few missense mutations were reported in the CCP1–4 region (43). Recently, a FH mutation in CCP1 associated with familial membranoproliferative glomerulonephritis (MPGN) (22) and one in CCP3 related with aHUS (44) were described. Thus, while mutations and autoantibodies affecting the FH C terminus are in general associated with aHUS, and mutations and autoantibodies affecting CCP1–4 are usually associated with C3G, the picture is not that simple (8, 10, 45, 46). N-terminal FH mutations causing alternative pathway dysregulation may be associated with different diseases. In addition, some identified gene variants may have no functional significance (47), or result in increased complement regulatory activity of FH (48). Therefore, it is important to characterize the mutant proteins in functional assays to identify those variants that have pathological relevance.

Previously, we reported novel aHUS-associated FH mutations, including a patient heterozygous for the N-terminal FH mutation c.592T > C leading to the W198R exchange (44). In the current study, we describe the functional characterization of this mutation that manifested in two different kidney diseases in the patient. The mutation is localized in the CCP3 domain and leads to functional FH deficiency, as we show that this mutation disrupts the main functions of the N-terminal portion of FH.

## Materials and Methods

The patient gave written informed consent in accordance with the Declaration of Helsinki. Written approval for the diagnostic tests and genetic analysis was given by the patient. Relevant clinical and laboratory data were collected from hospital records, according to the protocol approved by the institutional review board of Semmelweis University and the National Ethical Committee of Hungary.

### Generation of FH 1–4 Mutant

Codon-optimized FH CCPs 1–4 fragment (FH1–4) (49) was generated by commercial gene synthesis (GenScript, Piscataway, NJ, USA) and cloned into the pBSV-8His Baculovirus expression vector using PstI and SmaI enzymes, as previously described (50). Codon-optimized FH1–4 containing the W198R exchange was synthetized (IDT, Coralville, IA, USA) and cloned into the pBSV-8His vector using PstI and SmaI enzymes (50). The cloned constructs were verified by sequencing to ensure that the recombinant protein carried exclusively the W198R mutation. Both proteins were expressed in *Spodoptera frugiperda* (*Sf9)* insect cells using Insect-XPRESS™ Protein-free Insect Cell Medium (Lonza) and purified from the cell culture supernatants by affinity chromatography using an anti-FH column.

### Proteins, Abs, and Sera

Purified human FH, factor B (FB), factor D (FD), properdin (factor P), C3b, FI, goat anti-human FH Ab, and goat anti-human FB Ab were obtained from Merck (Budapest, Hungary). The anti-FH mAb A254 was purchased from Quidel (Biomedica; Budapest, Hungary), and mAb OX24 was from Santa Cruz Biotechnology. HRP-conjugated goat anti-human C3 was purchased from MP Biomedicals (Solon, OH, USA). HRP-conjugated rabbit anti-goat Ig and HRP-conjugated goat anti-mouse Ig were from Dako (Hamburg, Germany). Bovine serum albumin (BSA) was from Applichem (Darmstadt, Germany). Normal human plasma was collected from healthy individuals after informed consent and pooled.

### Sequence Analysis

Nucleotide and protein sequences were analyzed using the Bioinformatics Research Portal (ExPasy) of the Swiss Institute of Bioinformatics (http://www.expasy.org), to analyze the translation, molecular weight, and other parameters by the ProtParam tool. The position of the mutation was visualized using PyMol on the structure of the C3b in complex with FH1–4 (PDB ID 2WII) (51).

### C3b Binding Assays

To compare the binding of C3b to FH1–4_WT_ and FH1–4_W198R_ by ELISA, microtiter plate wells were coated with 100 nM of FH1–4_WT_ and FH1–4_W198R_ in Dulbecco’s PBS (DPBS; Lonza) at 20°C for 1 h. The wells were washed after each step with DPBS containing 0.05% Tween-20. After blocking with 5% BSA in DPBS-Tween for 1 h, serial dilution of C3b was added in DPBS at 20°C for 1 h. Bound C3b was detected with HRP-conjugated anti-C3 antibody, using 3,3′,5,5′-tetramethylbenzidine (TMB) (BioLegend; Biomedica, Budapest, Hungary) as chromogen and reading the absorbance at 450 nm.

The interaction of FH1–4_WT_ and FH1–4_W198R_ with C3b was also analyzed by surface plasmon resonance, using ProteOn XPR36 equipment (BioRad). Recombinant FH1–4_WT_ and FH1–4_W198R_ were diluted in acetate buffer (pH 4.0) and immobilized on two parallel flowcells on a GLC biosensor chip using a standard amine coupling protocol, as recommended by the manufacturer, to achieve a coupling density of ~3,500 RU for the two proteins. Serial dilutions of C3b were used as analytes, diluted in PBS containing 0.005% Tween-20 and 145 mM NaCl as running buffer at a flow rate of 30 µl/min. Association was followed for 300 s and the dissociation for 600 s. Data were analyzed with ProteOn manager software.

### C3bBb-Decay Assay

The C3bBb convertase was generated on C3b immobilized at 5 µg/ml in microtiter plate wells in DPBS, as described previously (52). Briefly, the wells were washed with DPBS containing 0.05% Tween-20 and blocked for 1 h at 20°C with DPBS containing 5% BSA. FB (2 µg/ml), FD (0.1 µg/ml), and properdin (4 µg/ml) were added in convertase buffer (4% BSA, 2 mM Ni^2+^, 0.1% Tween-20 in DPBS containing Ca^2+^ and Mg^2+^) in 50 µl for 30 min at 37°C. Decay acceleration was analyzed by adding increasing concentrations of FH1–4_WT_ and FH1–4_W198R_, 50 nM FH and 50 nM HSA for 60 min at 37°C. Remaining intact convertases were detected using polyclonal anti-FB antibody and the corresponding secondary antibody. Color reaction was developed with TMB and the absorbance was read at 450 nm.

### Cofactor Assay

Cofactor activity of FH1–4_WT_ and FH1–4_W198R_ was measured in fluid-phase by incubating 220 nM C3b and 440 nM FI with 10 and 50 nM FH, FH1–4_WT_ and FH1–4_W198R_ for 1 h at 37°C in a final volume of 28 µl. The reactions were stopped by adding reducing SDS-sample buffer. Samples were separated on 10% SDS-PAGE gel and subjected to Western blot. The membrane was blocked with DPBS containing 4% BSA and 1% skimmed milk powder. C3 fragments were detected using HRP-conjugated goat anti-human C3 antibody and an ECL detection kit (Merck).

### Hemolysis Assays

Sheep red blood cells (SRBCs; Culex Bt., Budapest, Hungary) were washed three times in veronal buffer containing 10 mM Mg^2+^-EGTA (Lonza; Biocenter, Szeged, Hungary). FH1–4_WT_ and FH1–4_W198R_ were added in 125–1,000 nM concentration to 2% SRBCs and 15% human serum with 1.5 µg mAb OX24 in a final volume of 60 µl in veronal buffer, and incubated at 37°C for 30 min. SRBCs were sedimented by centrifugation and the released hemoglobin was measured at 405 nm.

To determine the capacity of FH1–4_WT_ and FH1–4_W198R_ to reverse the alternative pathway-mediated lysis of rabbit red blood cells (RRBCs; kind gift of Á. Mikesy, Budapest), the recombinant proteins were added in 1 µM concentration to 2% RRBCs and 5% human serum in a final volume of 60 µl in veronal buffer containing 10 mM Mg^2+^-EGTA. Samples were incubated at 37°C for 30 min, and hemolysis was measured as above.

## Results

### Patient Description

In 2006, a 20-year-old man was admitted for evaluation of hematuria, proteinuria (protein/creatinine 0.83 g/g), and hypertension. He had a normal kidney function (creatinine 0.9 mg/dl), urine microscopy revealed dysmorphic hematuria. Laboratory analysis showed normal values for anti-nuclear antibodies, anti-neutrophil cytoplasmic antibodies, IgA, and complement C4. The only notable abnormality was a slightly decreased serum-complement C3c level (0.872 mg/ml; normal range: 0.9–1.8 mg/ml). The diagnosis of chronic glomerulonephritis was made. The patient was treated with an ACE-inhibitor and scheduled for short-term follow-up visits with the prospective of a renal biopsy in case of increasing proteinuria or worsening kidney function. Unfortunately, the patient did not reappear for the scheduled visits.

In 2012, the patient was admitted to our intensive care unit with end-stage renal disease, malignant hypertension, and pulmonary edema, and laboratory results consistent with TMA (platelet count 109 G/l, LDH 1,136 U/l, and haptoglobin not detectable). C3c was markedly decreased (0.49 mg/ml). The patient was started on hemodialysis. A diagnosis of aHUS was considered, but with better blood pressure control the laboratory parameters of TMA quickly normalized. The patient remained on chronic dialysis treatment. Because of a possible diagnosis of aHUS, and the fear of a relapse after transplantation and the need for eculizumab, the patient received full antimeningococcal vaccination.

In October 2013, the patient’s samples were sent to the 3rd Department of Internal Medicine, Semmelweis University, for further complement measurements during the diagnostic work-up, and the following concentrations were observed (with the reference ranges shown): 0.8 mg/ml of C3 (0.9–1.8 mg/ml), 400 mg/l of FH (250–880 mg/l), 114% of FI, and 63% of factor B (70–130% for the latter two, expressed in the percent of the normal human serum pool).

In March 2015, he received a full-house HLA-identical kidney graft from a deceased donor. Because of the assumption that the patient may have aHUS as underlying disease, calcineurin inhibitors were avoided and the initial immunosuppression consisted of belatacept, mycophenolic acid, and steroids. Despite optimal HLA-matching the patient suffered from delayed graft function for 3 weeks. A transplant biopsy showed severe acute cellular vascular rejection (BANFF 2b). The patient was treated with high-dose steroids and antithymocyte globulin and transplant function improved. 2 weeks later, a sudden elevation of LDH and decrease of serum haptoglobin were noted. Thrombocytes fell from 300G/l to 175G/l, and fragmentocytes were detected, suggestive of a diagnosis of TMA. The patient had severe hypertension. Assuming recurrence of aHUS, the patient was given a single dose of eculizumab (900 mg). However, an urgently performed second transplant biopsy showed marked narrowing of arterioles due to hyalinosis without any signs of TMA, therefore, eculizumab was not administered more than once. After consequent antihypertensive treatment all parameters of intravascular hemolysis resolved. The further posttransplant course was unremarkable and the patient’s current serum creatinine is 1.7 mg/dl corresponding to an estimated glomerular filtration rate of 51 ml/min/1.73 m^2^.

The patient’s current immunosuppression consists of belatacept 250 mg every 4 weeks, mycophenolate sodium 720 mg and prednisolone 5 mg. Antihypertensive medication includes valsartan, amlodipin, doxazosin, metoprolol, and hydrochlorothiazide.

**Figure 1:**
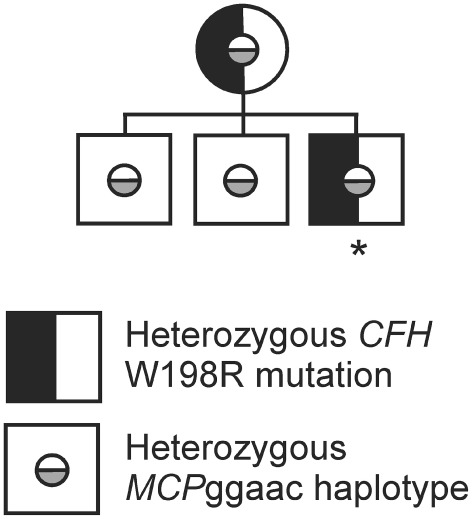
Family tree of the patient’s family. The family tree shows the occurrence of the complement factor H (*CFH*) W198R mutation in heterozygosis in the mother and the index patient. All investigated family members carry the membrane cofactor protein risk haplotype (*MCP*ggaac) in heterozygosis. The patient is indicated with an asterisk, all other family members are healthy (44).

While screening for mutations, the whole coding regions of the genes encoding complement factor H (*CFH*), factor I (*CFI*), membrane cofactor protein (*CD46*), thrombomodulin, factor B (*CFB*), and C3 (*C3*) were analyzed by direct DNA sequencing following PCR amplification. Upon genetic analysis, the membrane cofactor protein risk haplotype (MCPggaac) (53) was identified in the patient, his mother, and two brothers in heterozygosis. The patient and his mother are heterozygous for the FH mutation W198R, as we reported previously (44). Her serum C3 level was slightly reduced (0.82 mg/ml). However, she had no sign of kidney disease. The two brothers of the patient do not carry the FH W198R mutation (we did not receive samples to measure the serum C3 levels in the two brothers) (Figure 1) (44).

### Generation of FH1–4_WT_ and FH1–4_W198R_

**Figure 2:**
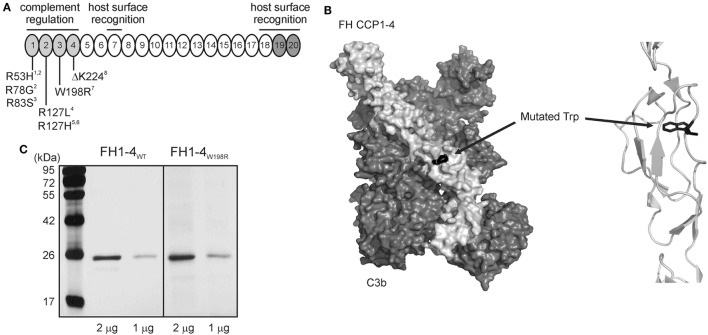
Location of W198R in FH, and generation of mutant FH1-4 fragment. **(A)** FH has 20 complement control protein domains (CCPs). The N-terminal CCPs 1–4 are responsible for complement regulation (light gray), and the CCPs 19–20 are host surface recognition domains (dark gray). The position of the mutation is showed with other previously characterized mutations. W198R is located in CCP3. References: 1 (54), 2 (55), 3 (22), 4 (42); 5 (56), 6 (57), 7 (44), 8 (20). **(B)** *Left*, 3D structure of the mutant FH CCPs 1–4 (light gray) with C3b (dark gray) based on the known co-crystal structure of FH CCPs 1–4 and C3b (PDB 2WII) (51) (visualized using PyMol). The surface representation of the residues is shown. *Right*, close-up view of the FH region containing the mutated residue shown as cartoon representation. The Trp residue, shown in black as a stick model, is only partially exposed to the surface, opposite to the one in contact with C3b. The substitution to Arg is expected to perturb the local fold, since the Trp side chain is oriented to the interior of the domain. **(C)** Recombinant mutant FH1–4 was expressed in insect cells. 1 and 2 µg of the purified wild-type and mutant FH CCPs 1–4 were run on 12% SDS-PAGE and stained with silver nitrate. The molecular mass marker is shown on the left side (kDa).

In order to assess the functional impact of the W198R mutation, we generated a wild-type (FH1–4_WT_) and a mutant form (FH1–4_W198R_) of the complement regulatory domains of FH that were expressed as recombinant proteins in insect cells. The W198R mutation is located in one of the regulatory domains (CCP3) and affects the conserved Trp residue of the CCP domain (Figure 2A). The substitution is expected to perturb the local fold, since the Trp side chain is oriented to the interior of the domain and maintains its structure (Figure 2B). However, we did not observe a secretion defect or instability of the recombinant protein (Figure 2C). In line with this, the patient had normal FH level (400 µg/ml), thus the mutation is unlikely to lead to partial FH deficiency due to unstable protein produced by the affected allele.

### W198R Impairs C3b Binding of FH

As the 3D structure of the FH CCPs 1–4 with C3b shows (Figure 2B), the Trp residue is only partially exposed to the surface, opposite to the one in contact with C3b. The introduction of a positive charge at this place would result in the disruption of the local structure. Therefore, even if the residue is at the opposite surface and not a contact one with C3b, the structural change could be strong enough to cause a loss of C3b binding.

**Figure 3:**
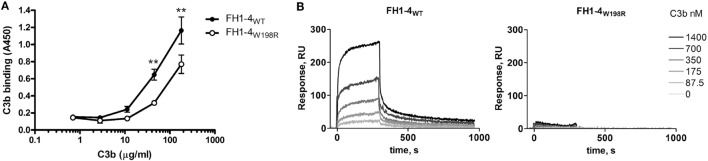
Analysis of C3b-binding capacity of FH1–4_W198R_ by ELISA and SPR. **(A)** Both the wild-type and the mutant proteins were immobilized in microtiter plate wells at 100 nM. Serial dilutions of C3b were added and its binding was detected with anti-C3 antibody. Data are mean ± SD from three experiments. The binding of C3b to FH1–4_W198R_ was significantly weaker than to FH1–4_WT_ (*p* = 0.0003 two-way ANOVA; **p < 0.01). **(B)** SPR analysis of the interaction of the immobilized FH1–4_WT_ and FH1–4_W198R_ with serial dilutions of C3b in the fluid phase. PBS-0.005% Tween 20 was used as a running buffer, with a flow rate of 30 µl/min.

To test if the mutation affects binding of the main ligand C3b, we immobilized FH1–4_WT_ and FH1–4_W198R_ in equimolar amounts in microplate wells. Increasing concentrations of purified C3b were added, and bound C3b was detected with a polyclonal antibody in ELISA. Both the wild-type and the mutant FH1–4 bound C3b in a dose-dependent manner; however, the capability of FH1–4_W198R_ to bind C3b was significantly reduced compared with FH1–4_WT_ (Figure 3A). The interaction with C3b was also analyzed by surface plasmon resonance. The FH1–4_W198R_ and FH1–4_WT_ were immobilized on the chip surface and C3b was flowed over the cells. In this set-up, the FH1–4_W198R_ practically showed no C3b binding (Figure 3B). Altogether, these results indicated that the mutation results in a strongly reduced capacity of FH to bind C3b *via* its N terminus.

### W198R Impairs Cofactor and Decay Accelerating Activity of FH

**Figure 4:**
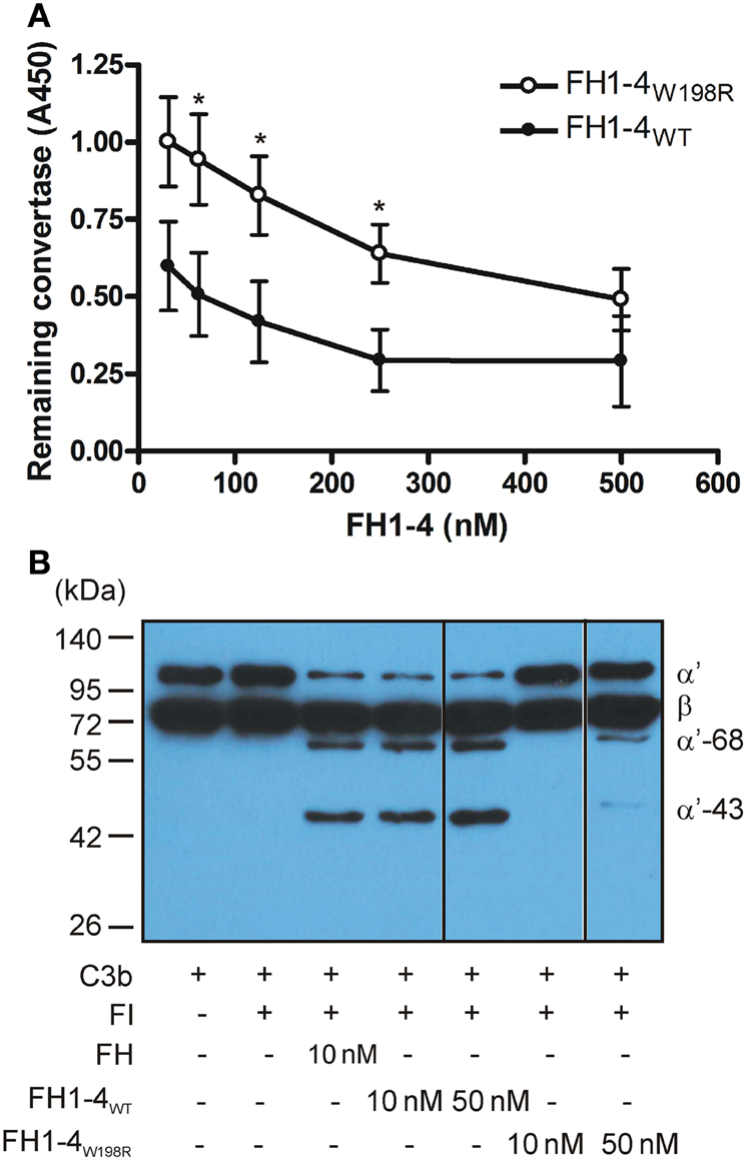
Decay accelerating- and cofactor activity of FH1–4_W198R_. **(A)** Decay acceleration assay. The alternative pathway C3 convertase (C3bBb) was built up on microtiter plate. FH1–4_WT_ and FH1–4_W198R_ were added for 1 h, and the remaining intact convertase was detected using anti-factor B antibody. The capacity of FH1–4_W198R_ to accelerate convertase decay was significantly impaired compared with FH1–4_WT_ (*p* < 0.0005, two-way ANOVA; *p < 0.05). Data are mean ± SD from three experiments. **(B)** The cofactor activity of FH1–4_WT_ and FH1–4_W198R_ in the fluid phase was measured by incubating 220 nM C3b and 440 nM FI with the indicated concentrations of FH1–4_WT_ and FH1–4_W198R_ at 37°C for 1 h. To stop the reaction, reducing sample buffer was added, and the samples were subjected to 10% SDS-PAGE and Western blotting. To visualize the C3b proteolysis, HRP-conjugated polyclonal anti-human C3 antibody was used. The blot is representative of two experiments.

Because of the reduced C3b binding, we examined whether this mutation influences the regulatory functions of FH. First, we tested the decay of the solid-phase alternative pathway C3 convertase (C3bBb) assembled on an ELISA plate. Increasing concentrations of the mutant and wild-type FH1–4 fragments were added, and the remaining convertase was monitored using a factor B-specific antibody. While both FH1–4_W198R_ and FH1–4_WT_ dissociated the convertase in a dose-dependent manner, the mutant form had significantly less capacity to accelerate convertase decay (Figure 4A).

Next, we tested the effect of the mutation on the FH cofactor activity in fluid phase. C3b cleavage by the serine protease FI in the presence of equimolar amounts of FH1–4_W198R_, FH1–4_WT_, and FH as control, was measured by detecting the generated C3b fragments by Western blot. At 10 nM concentration, both FH and FH1–4_WT_ acted efficiently as cofactors for FI, demonstrated by the cleavage of the C3b α′ chain into 68- and 43-kDa fragments. Under these conditions, when FH1–4_W198R_ was added as cofactor, no C3b cleavage was apparent (Figure 4B). At increased, 50 nM concentration FH1–4_W198R_ displayed weak cofactor activity that was still strongly reduced compared with the activity of 10 nM FH1–4_WT_.

Altogether, these data demonstrate that the W198R exchange results in reduced binding of fluid-phase C3b and, as a consequence, reduced fluid-phase cofactor activity and surface-bound convertase decay accelerating activity of FH.

### W198R Causes Reduced Protection of Host-Like Cells from Complement-Mediated Lysis

**Figure 5:**
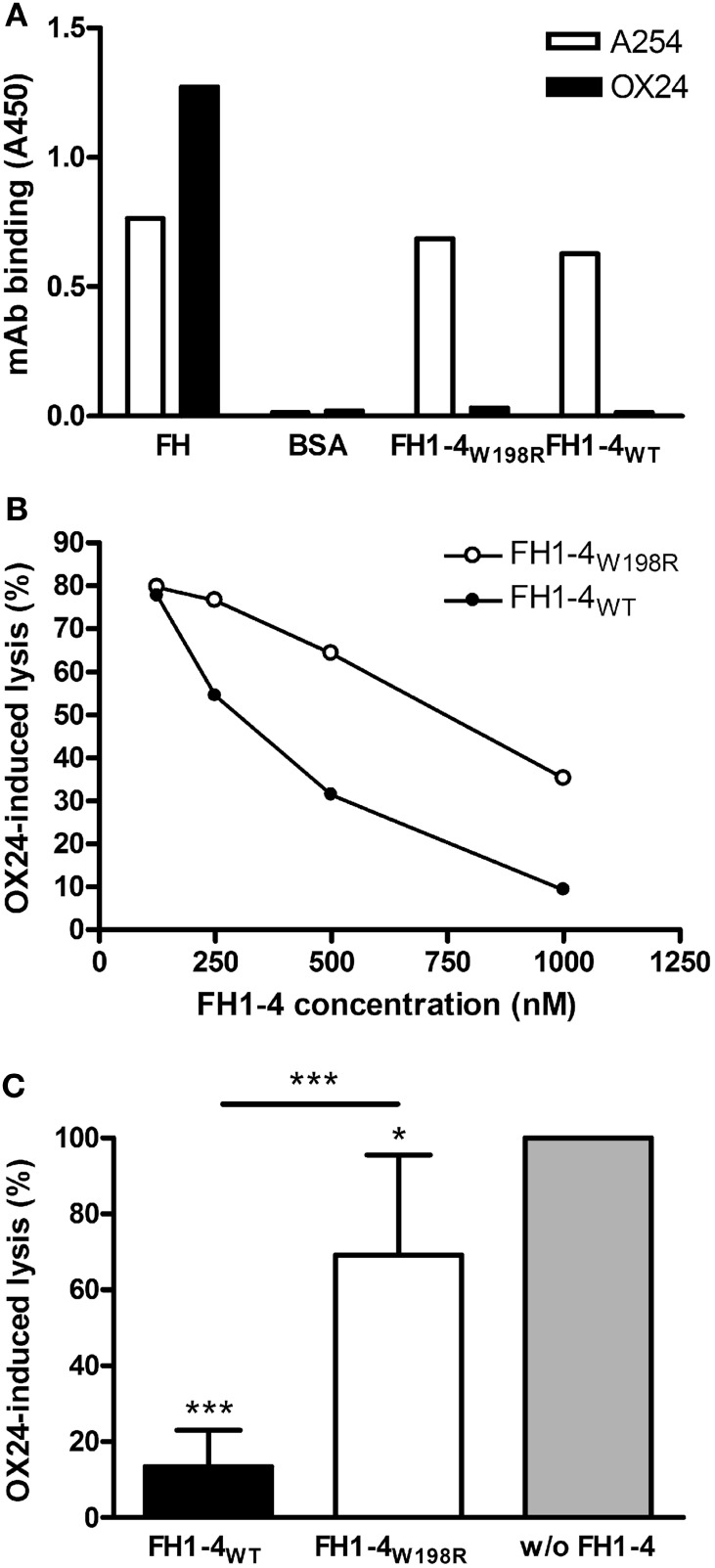
Impaired capacity of FH1–4_W198R_ to protect sheep red blood cells (SRBCs) from complement-mediated lysis. **(A)** The binding of mAb OX24, which was used to induce SRBC lysis, to immobilized FH1–4_WT_ and FH1–4_W198R_, as well as to factor H (FH) and bovine serum albumin (BSA), used as positive and negative controls, respectively, was measured in ELISA. Reactivity of the mAb A254 that binds in CCP1 of FH is also shown as a control. A representative ELISA result is shown. **(B)** 15% normal human serum was mixed with 1.5 µg OX24 antibody, which binds in FH CCP5, to induce lysis of SRBCs. Samples included increasing concentrations of FH1–4_WT_/FH1–4_W198R_. After incubating at 37°C for 30 min, the released hemoglobin in the supernatants was measured at 405 nm. A representative experiment is shown. **(C)** To quantify and compare the capacity of wild-type and mutant FH1–4 at high concentration to inhibit complement activation in serum, OX24-induced SRBC lysis was measured in the presence of 1 µM FH1–4_WT_ and FH1–4_W198R_. SRBC lysis in the sample without the addition of FH1–4 was set to 100%. Data are mean + SD of four independent experiments (*p < 0.05, ***p < 0.001, one-way ANOVA).

To analyze the effect of the mutation on cellular protection against complement activation, various hemolysis assays were used. Hemolysis assays using sheep red blood cells (SRBCs) are commonly used to evaluate the capacity of FH to protect host-like cells from complement-mediated lysis, because—similar to human cells—FH can bind to surface exposed polyanionic molecules, likely sialic acid, on the surface of SRBCs (58). First, we induced lysis of SRBCs with the OX24 mAb, which binds to CCP5 of FH and inhibits the complement regulatory activity of FH in serum but also when FH is bound to the cells. We confirmed in ELISA that this mAb does not bind to the generated wild-type and mutant FH1–4 (Figure 5A). To inhibit the complement activation in the fluid-phase and thus prevent bystander lysis of SRBCs, increasing concentrations of FH1–4_W198R_ and FH1–4_WT_ were added. Both FH1–4 constructs reduced SRBC lysis in a dose-dependent manner; however, in this functional assay, the FH1–4_W198R_ was less effective to prevent hemolysis than the wild-type protein (Figure 5B). Even when added in 1 µM concentration, FH1–4_W198R_ could only slightly reduce hemolysis, which activity was significantly weaker than that of FH1–4_WT_ (Figure 5C).

**Figure 6:**
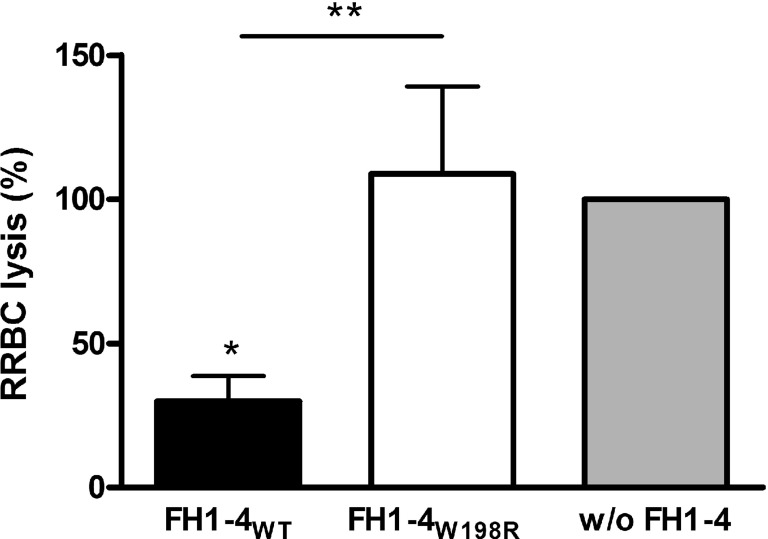
Impaired capacity of FH1–4_W198R_ to protect rabbit red blood cells (RRBCs) from human complement-mediated lysis. RRBCs were incubated with 5% normal human serum. RRBCs cannot bind FH because of the lack of sialic acids on their surface; therefore, they are lysed by serum complement. FH added to the serum in excess can inhibit complement activation and reduce complement-mediated lysis of RRBCs. To quantify and compare the capacity of FH1–4_WT_ and FH1–4_W198R_ to inhibit complement activation in serum, FH1–4_WT_/FH1–4_W198R_ were added in 1 µM concentration, the samples were incubated at 37°C for 30 min, then the released hemoglobin in the supernatants was measured at 405 nm. RRBC lysis in serum without the addition of FH1–4 was set to 100%. Data are mean + SD of three independent experiments (*p < 0.05, **p < 0.01, one-way ANOVA).

We also examined the alternative pathway mediated lysis of rabbit erythrocytes (RRBCs). Because FH cannot bind to rabbit erythrocytes due to the lack of sialic acids on their surface, normal human serum causes dose-dependent lysis of RRBCs. In this assay, FH1–4_W198R_ when added at 1 µM concentration did not protect the RRBCs from complement-mediated lysis, whereas FH1–4_WT_ strongly inhibited RRBC lysis (Figure 6).

## Discussion

The kidney diseases associated with complement alternative pathway dysregulation may represent a spectrum of related disorders, with overlapping underlying genetic or acquired factors identified in distinct disease entities (7, 8). It is important to functionally characterize the identified genetic changes, because they may represent polymorphisms with no functional significance (47). In this study, we describe the functional consequences of the FH W198R mutation by comparing the recombinant wild-type and mutant complement regulatory domains (i.e., FH1–4). This missense mutation results in a strong reduction of the complement regulatory activity.

The soluble complement inhibitor FH plays an important role in both plasma and at cellular and non-cellular surfaces, such as the glomerular basement membrane in the kidney. FH deficiency and thus uncontrolled alternative pathway activation lead to various diseases from aHUS to C3G. Very low level, complete absence in plasma or functional inactivation of FH result in continuous C3-fragment deposition in the glomeruli and usually manifest as C3G. The W198R mutation does not lead to quantitative FH deficiency, because the serum FH level was normal in the patient. However, a partial functional FH deficiency is evident from the *in vitro* characterization of the mutant protein (Figures 3–6). Previous *in silico* analysis predicted a deleterious effect of the mutation, and a hemolytic test indicated abnormally, but not dramatically, elevated hemolysis of SRBCs in the presence of the patient’s serum (44). In the current study, we found strongly reduced C3b binding in the case of the mutant protein (Figure 3). Compared to the ELISA, the SPR approach showed complete lack of C3b binding for FH1–4_W198R_. This difference may be due to the different surface protein densities in the two assays, the different buffers (while the ionic strength was similar, in the SPR experiments the buffer contained 0.005% Tween-20), and the short (300 s) contact time in the SPR versus long incubation time (1 h) in the ELISA. The longer incubation and steady state condition in the ELISA allow the formation of detectable amount of the low affinity FH1–4_W198R_—C3b complexes, which is not supported by the stringent conditions in the SPR measurement. In addition, in the ELISA, the detection of the formed complexes is enhanced by an additional step using the anti-C3 antibody and by the enzyme conjugated to the antibody. In any case, a significant reduction of C3b binding by FH1–4_W198R_ in comparison with FH1–4_WT_ was evident, which translated into a dramatic impairment of complement regulation by FH in both fluid-phase and solid-phase assays, as well as in cell surface protection assays.

The mutation is located at the surface of CCP3, opposite to the contact interface with C3b (Figure 2B). The decreased C3b binding could be explained by a major conformational change, affecting the entire domain, caused by the replacement of a large aromatic aminoacid with a charged one. The profound perturbation of the FI cofactor activity could be a synergistic effect between decreased C3b binding and decreased binding of FI to the C3b-mutant FH complex, since the mutation is located very close to the FI binding site in CCP3 of FH (59).

This N-terminal FH mutation manifested sequentially as glomerulonephritis (possibly C3G) and then aHUS in the same patient. The patient is heterozygous for the W198R mutation; he had normal FH serum levels and it was not possible to determine whether the wild-type and mutant FH alleles were expressed differently. Our previous results of SRBC hemolysis in the serum of the patient (44) indicates that both FH variants are expressed, and the recombinant protein was also expressed and stable (Figure 2C). It is likely that the reduced amount of functionally active FH would not be able to cope with too strong complement activation in plasma, and the balance would be tipped in favor of activation instead of inhibition. This mutation is in the FH N terminus and not in the C-terminal domains CCPs 18–20 that are mainly responsible for host surface recognition and implicated in aHUS, thus both the mutant and the wild-type FH in the patient could bind to surfaces such as glomerular endothelial cells and the basement membrane. However, due to the affected regulatory domains in the mutant protein, this would result in suboptimal surface protection against complement attack that could be overwhelmed under conditions of too strong complement activation.

It is notable that our patient presented in 2012 with malignant hypertension and moderate hematologic activity of TMA. A subset of patients with hypertension-associated TMA falls within the spectrum of complement-mediated TMA (60), and our patient is an example of this. Phenotype transitions in patients have been described earlier, such as mesangio-capillary glomerulonephritis with initial presentation of aHUS (61), and MPGN with transition to aHUS associated with FH mutations R53C (CCP1) and I216T (CCP4) (62, 63).

To date, only a few N-terminal FH mutations were characterized in detail. A homozygous deletion of K224 in CCP4 resulted in a functionally inactive FH and caused dense deposit disease (20). The heterozygous R83S mutation in CCP1 was shown to lead to impaired C3b binding, reduced cofactor, and decay accelerating activities and was associated with MPGN with acquired partial lipodystrophy (22). Two mutations affecting FH CCP1 R53H and R78G, both reported in aHUS patients and the latter in homozygosis, were characterized, and similarly defective complement regulating capacity of the mutants were found (54, 55). The homozygous R127L mutation in FH CCP2 was described in two brothers with undetectable serum FH levels who developed MPGN (42). Similarly, the homozygous R127H mutation caused complete lack of circulating FH in a boy with severe recurrent pneumonia but without renal manifestation; the mutant protein was later characterized and shown that albeit the mutant FH had normal cofactor activity, it was retained intracellularly (56, 57).

In summary, our results demonstrate that the FH W198R exchange results in impaired complement alternative pathway regulation. The heterozygous nature of this mutation in the index patient may explain the manifestation of two complement-mediated kidney diseases several years apart, likely due to different triggers leading to complement dysregulation in plasma or on cell surfaces.

## Ethics Statement

The patient gave written informed consent in accordance with the Declaration of Helsinki. Written approval for the diagnostic tests and genetic analysis was given by the patient. Relevant clinical and laboratory data were collected from hospital records, according to the protocol approved by the institutional review board of Semmelweis University and the National Ethical Committee of Hungary.

## Author Note

Parts of this work were presented at the 26th International Complement Workshop, September 4–8, 2016, Kanazawa, Japan (Immunobiology, 221: 1176).

## Author Contributions

ZP and MJ designed and supervised the study. MC and BU generated recombinant proteins. MC performed ELISA, western blot, functional and hemolysis assays. NSM carried out surface plasmon resonance analysis. DC performed genetic analyses. DC, EM, KL, and ZP collected clinical and laboratory data. MC and MJ wrote the manuscript with the help of the other authors. All authors revised and approved the manuscript.

## Conflict of Interest Statement

The authors declare that the research was conducted in the absence of any commercial or financial relationships that could be construed as a potential conflict of interest.

## Abbreviations

aHUS, atypical hemolytic uremic syndrome; C3G, C3 glomerulopathy; CCP, complement control protein domain; FH, factor H; FI, factor I; TMA, thrombotic microangiopathy.
